# [Arg^6^, D-Trp^7,9^, N^me^Phe^8^]-substance P (6–11) (antagonist G) inducesP-1 transcription and sensitizes cells to chemotherapy

**DOI:** 10.1054/bjoc.2000.1362

**Published:** 2000-09-04

**Authors:** A C MacKinnon, C Waters, I Rahman, N Harani, R Rintoul, C Haslett, T Sethi

**Affiliations:** Rayne Laboratory, Respiratory Medicine Unit, University of Edinburgh Medical School, Teviot Place, Edinburgh, EH8 9AG; Imperial Cancer Research Fund, Medical Oncology Unit, Western General Hospital, Crewe Road South, Edinburgh, EH4 2XU, UK

**Keywords:** SCLC, [Arg6, D-Trp7,9, NmePhe8]-substance P (6–11), antagonist G, AP-1, apoptosis, ROS, chemosensitivity

## Abstract

[Arg^6^, D-Trp^7,9^, N^me^Phe^8^]-substance P (6–11) (antagonist G) inhibits small cell lung cancer (SCLC) growth and is entering Phase II clinical investigation for the treatment of SCLC. As well as acting as a neuropeptide receptor antagonist, antagonist G stimulates c-jun-N-terminal kinase (JNK) activity and apoptosis in SCLC cells. We extend these findings and show that the stimulation of JNK and apoptosis by antagonist G is dependent upon the generation of reactive oxygen species (ROS) being inhibited either by anoxia or the presence of N-acetyl cysteine (n-AC). Antagonist G is not intrinsically a free radical oxygen donor but stimulates free radical generation specifically within SCLC cells (6.2-fold) and increases the activity of the redox-sensitive transcription factor AP-1 by 61%. In keeping with this, antagonist G reduces cellular glutathione (GSH) levels (38% reduction) and stimulates ceramide production and lipid peroxidation (112% increase). At plasma concentrations achieved clinically in the phase I studies, antagonist G augments, more than additively, growth inhibition induced by etoposide. Our results suggest that antagonist G may be particularly effective as an additional treatment with standard chemotherapy in SCLC. These novel findings will be important for the clinical application of this new and exciting compound and for the future drug development of new agents to treat this aggressive cancer. © 2000 Cancer Research Campaign


*British Journal of Cancer* (2000) **83**(7), 941–948
© 2000 Cancer Research Campaign
doi: 10.1054/ bjoc.2000.1362, available online at http://www.idealibrary.com on IDEAL®

### [Arg$^6$, D-Trp$^{7,9}$, N$^{me}$Phe$^8$]-substance P (6–11) (antagonist G) induces AP-1 transcription and sensitizes cells to chemotherapy

**AC MacKinnon[1], C Waters[2], I Rahman[1], N Harani[1], R Rintoul[1], C Haslett[1] and T Sethi[1]**

[1]Rayne Laboratory, Respiratory Medicine Unit, University of Edinburgh Medical School, Teviot Place, Edinburgh, EH8 9AG; [2]Imperial Cancer Research Fund, Medical Oncology Unit, Western General Hospital, Crewe Road South, Edinburgh, EH4 2XU, UK

**Summary** [Arg$^6$, D-Trp$^{7,9}$, N$^{me}$Phe$^8$]-substance P (6–11) (antagonist G) inhibits small cell lung cancer (SCLC) growth and is entering Phase II clinical investigation for the treatment of SCLC. As well as acting as a neuropeptide receptor antagonist, antagonist G stimulates c-jun-N-terminal kinase (JNK) activity and apoptosis in SCLC cells. We extend these findings and show that the stimulation of JNK and apoptosis by antagonist G is dependent upon the generation of reactive oxygen species (ROS) being inhibited either by anoxia or the presence of N-acetyl cysteine (n-AC). Antagonist G is not intrinsically a free radical oxygen donor but stimulates free radical generation specifically within SCLC cells (6.2-fold) and increases the activity of the redox-sensitive transcription factor AP-1 by 61%. In keeping with this, antagonist G reduces cellular glutathione (GSH) levels (38% reduction) and stimulates ceramide production and lipid peroxidation (112% increase). At plasma concentrations achieved clinically in the phase I studies, antagonist G augments, more than additively, growth inhibition induced by etoposide. Our results suggest that antagonist G may be particularly effective as an additional treatment with standard chemotherapy in SCLC. These novel findings will be important for the clinical application of this new and exciting compound and for the future drug development of new agents to treat this aggressive cancer. © 2000 Cancer Research Campaign

**Keywords**: SCLC; [Arg$^6$, D-Trp$^{7,9}$, N$^{me}$Phe$^8$]-substance P (6–11); antagonist G; AP-1; apoptosis; ROS; chemosensitivity

Small cell lung cancer (SCLC), is a particularly aggressive form of lung cancer, it metastasizes widely and early and many patients have widespread metastases at presentation, precluding curative surgery (Ihde, 1992). Despite initial sensitivity to radio and chemotherapy SCLC almost invariably relapses, so that the 2-year survival remains less than 5% (Smyth et al, 1986). Novel forms of treatment are therefore urgently required.

SCLC cell proliferation is driven by multiple autocrine and paracrine growth loops involving calcium-mobilizing neuropeptides, including bradykinin, cholecystokinin, galanin, gastrin, gastrin releasing peptide (GRP), neurotensin and vasopressin (Moody et al, 1981; Cuttitta et al, 1985; Sethi and Rozengurt, 1991). Interruption of the mitogenic second messenger signals initiated by these neuropeptides could offer a novel and effective form of treatment to prevent SCLC cell growth. [D-Arg$^6$, D-Trp$^{7,9}$, N$^{me}$Phe$^8$]-substance P (6–11) (antagonist G) is a novel anti-cancer agent which is entering phase II clinical trials for the treatment of SCLC (Langdon et al, 1994; Jonkman-de Vries et al, 1998). Antagonist G belongs to a family of substance P-related peptides which inhibit the growth of SCLC cells in vitro and in vivo (Woll and Rozengurt, 1990; Sethi et al, 1992). Early studies in Swiss 3T3 cells showed that antagonist G and related substance P analogues inhibit the binding of neuropeptides to their receptors (Woll and Rozengurt 1988; Mitchell et al, 1995; Seckl et al, 1995), thus inhibiting neuropeptide stimulated mitogenic signalling (Mitchell et al, 1995; Seckl et al, 1995; 1996; 1997). However, recently it has been shown that in addition to inhibiting neuropeptide responses, antagonist G, and its analogue antagonist D, also have important agonist activity, activating c-jun N terminal kinase (JNK) in a neuropeptide-independent manner (Jarpe et al, 1998*a*; MacKinnon et al, 1999). These studies provide the molecular basis to understanding the mechanisms by which antagonist G inhibits both basal and neuropeptide-stimulated SCLC growth (Sethi et al, 1992; Langdon et al, 1994).

Apoptosis is an active cell-death programme which is initiated via specific signal transduction cascades (Kerr et al, 1972; Kyprianou et al, 1992). Factors affecting the balance between SCLC cell proliferation and apoptosis will have a profound effect on tumour growth. The stress-activated protein kinases or c-jun N-terminal kinases (JNKs) have been shown to be activated in response to apoptotic stimuli in several cell types (Verheij et al, 1996; Le-Niculescu, 1999). Previous studies have shown that expression of a dominant negative JNK1 mutant in SCLC cells inhibits UV-induced apoptosis, suggesting an essential role of JNK1 in the induction of apoptosis in SCLC cells (Butterfield et al, 1997). Our previous work has shown that antagonist G activates JNK1 in SCLC cells (MacKinnon et al, 1999).

The role of reactive oxygen species (ROS) in pathways leading to apoptosis is particularly pertinent in cancers where the oxygen tension at the centre of tumours may be particularly low (Bush et al, 1978). Although the exact role of ROS in the induction of apoptosis is unclear, it is becoming well established that ROS may be involved in the relay of early apoptotic signals by acting as signalling intermediates (Lo and Cruz, 1995). Recent evidence

*Received 12 January 2000*
*Revised 8 May 2000*
*Accepted 11 May 2000*

*Correspondence to*: AC MacKinnon suggests that different forms of chemotherapy primarily exert their cytotoxic effects by inducing apoptosis (Friesen et al, 1999). The generation of intracellular ROS and/or the depletion of intracellular antioxidants may sensitize tumours to the pro-apoptotic effects of chemotherapeutic agents (Carmichael et al, 1988).

The aim of this study was to define the molecular mechanisms underlying these observations and their relevance to the use of antagonist G in clinical practice. Our results show that antagonist G induces ROS generation and stimulates lipid peroxidation while reducing GSH levels. This is cell type-specific and plays an important part in the apoptotic effect of antagonist G. In addition we show that antagonist G sensitizes SCLC cells to chemotherapeutic agents. These findings have important implications in the future clinical use and development of antagonist G and in the design of novel therapeutic agents to treat this aggressive cancer.

### MATERIALS AND METHODS

### Materials

SCLC cell lines NCI-H69 and NCI-H510 and CHO-K1 cells were purchased from the American Type Tissue Culture Collection (Rockville, MD, USA); RPMI-1640, dihydrorhodamine and etoposide from Sigma (Poole, Dorset, UK); annexin V-FITC from Bender Medsystems (UK); antagonist G ([$Arg^6$, D-$Trp^{7,9}$, $N^{me}Phe^8$]-substance P (6–11)) was a kind gift from Peptec (Copenhagen, Denmark). All other reagents were of the purest grade available.

### Cell culture

NCI-H69 and NCI-H510 SCLC cells were cultured in RPMI 1640 medium with 25 mM HEPES supplemented with 10% (v/v) fetal bovine serum, 50 U $ml^{-1}$ penicillin, 50 μg $ml^{-1}$ streptomycin and 5 μg $ml^{-1}$ L-glutamine in a humidified atmosphere of 5% $CO_2$/95% air at 37°C. For experimental purposes, the cells were grown in SITA medium consisting of RPMI 1640 medium supplemented with 30 nM selenium, 5 μg $ml^{-1}$ insulin, 10 μg $ml^{-1}$ transferrin and 0.25% (w/v) BSA. Cells were quiesced in serum-free RPMI 1640 medium containing 0.25% (w/v) BSA. In anoxic studies, cells were incubated at 37°C in a MK3 anaerobic incubator with 0% oxygen (Don Whitely Scientific Ltd, Yorkshire, UK). CHO-K1 cells were maintained in Dulbecco's modified Eagles medium (DMEM) containing 10% (v/v) fetal calf serum, 50 U $ml^{-1}$ penicillin, 50 μg $ml^{-1}$ streptomycin and 5 μg $ml^{-1}$ L-glutamine.

### Measurement of apoptosis

SCLC cells ($10^6$ cells $ml^{-1}$) were incubated in quiescent medium in the presence or absence of antagonist G or other test agents for 24 h. CHO-K1 cells were incubated with antagonist G in DMEM containing 0.1% fetal bovine serum for 24 h.

#### *Morphology*

Cells were cytocentrifuged onto glass slides, fixed with methanol, stained with May–Grünwald–Giemsa stain and examined using an Olympus BH-2 microscope, at a magnification of × 400. Apoptotic SCLC cells displayed typical morphological features, including cell shrinkage and chromatin condensation as previously described (Tallet et al, 1996).

#### *Annexin V binding*

H69 cells and trypsinized CHO-K1 cells were washed in PBS and incubated with annexin V-FITC (1:250) for 10 min at 4°C then fixed in 3% paraformaldehyde. The percentage of annexin V positive cells were determined by flow cytometry using an EPICS Profile II flow cytometer (Coulter Electronics, Luton, UK).

#### *DNA laddering*

DNA ladders from antagonist G-treated cells were prepared as described (Tallet et al, 1996).

### Measurement of intracellular reactive oxygen species (ROS)

Intracellular ROS were measured by dihydrorhodamine fluorescence. H69 SCLC cells and trypsinized CHO-K1 cells were washed and incubated ($10^6$ cells $ml^{-1}$) with 1 μM dihydrorhodamine (DHR) for 5 min at 37°C in phosphate buffered saline (PBS) prior to the addition of antagonist G or $H_2O_2$ for a further 10 min at 37°C. Fluorescence was measured by flow cytometry using an EPICS Profile II flow cytometer (Coulter Electronics, Luton, UK).

### Measurement of c-jun-N terminal kinase (JNK)

Quiesced H69 cells were washed twice in PBS (pH 7.4), and incubated for various times with test agents as indicated in the figure legends. Following immunoprecipitation of JNK1 from whole cell lysates, kinase activity was carried out as described (Coso et al, 1995; MacKinnon et al, 1999) 1 μg GST-c-jun(79) substrate. Phosphorylated c-jun was identified from autoradiographs of Coomasie Blue stained-SDS-PAGE gels, and quantified by optical densitometry.

### Measurement of AP-1 activity

H69 cells were quiesced overnight in serum-free medium, washed and incubated ($10^7$ cells $ml^{-1}$) with test agents for 30 min at 37°C. Nuclear extracts were prepared by the method of Staal et al (1990). The oligonucleotides for AP-1 (Promega) were end-labelled using T4 polynucleotide kinase and [$\gamma^{32}$P]-ATP (Promega). Binding reactions were carried out using 5 μg nuclear extract protein, 0.25 mg $ml^{-1}$ poly (dI-dC).poly (dI-dC) (Pharmacia Biotech, St. Albans, UK), in 20 μl binding buffer (Promega). Competition studies were carried out using a 100-fold excess of either unlabelled AP-1 oligonucleotide (competitor) or unlabelled NFκβ oligonucleotide (non-competitor). The protein complexes were resolved on 6% non-denaturing polyacrylamide gels and detected by autoradiography.

### Measurement of lipid peroxidation and cellular glutathione levels

SCLC cells ($10^7$ cells $ml^{-1}$) in serum-free media were incubated with antagonist G for 24 h. Lipid peroxides were assayed as thiobarbituric acid reactive substances (TBARS) as previously described (Ohkawa et al, 1979). Following precipitation of proteins with 0.1% (v/v) Triton X-100 and 0.6% (w/v) sulphosalicylic acid, total cellular glutathione was measured as described (Tietze, 1969).

 © 2000 Cancer Research Campaign

### Ceramide quantitation

SCLC cells ($10^7$ cells $ml^{-1}$) in SITA medium were treated with test agents as indicated and the lipids extracted (Bligh et al, 1959) and dried under vacuum. Ceramide was quantitated using the diacylglycerol (DAG) kinase assay as previously described (Jayadev et al, 1994).

### Chemosensitivity

SCLC cells, 3–5 days post-passage, were washed and resuspended in SITA medium at a density of $5 \times 10^4$ cells $ml^{-1}$ in the presence of 0–100 μg $ml^{-1}$ etoposide with or without antagonist G. At each time-point cell number was determined using a Coulter counter (Coulter Electronics).

### RESULTS

### Antagonist G-induced apoptosis is redox-sensitive and caspase-dependent

Antagonist G caused a marked concentration-dependent stimulation of apoptosis in SCLC cells as judged morphologically. In the SCLC cell line H69, antagonist G (25 μM) increased basal apoptosis from 10.5 ± 4.2% to 37.1 ± 5.4% (*n* = 4), with an $EC_{50}$ of 5.9 ± 0.1 μM (*n* = 4, Figure 1A). Antagonist G also induced apoptosis in the SCLC cell line H510 with an $EC_{50}$ value of 15.2 ± 2.7 μM (Figure 1A). Apoptosis was confirmed using DNA laddering (Figure 1C) and annexin V binding (Figure 2) and was first apparent after only 6 h incubation (data not shown). The effect of 50 μM antagonist G on apoptosis was inhibited by co-incubation with Z-Val-Ala-DL-Asp fluormethylketone (ZVAD-fmk) (Figure 1C) from 36.5 ± 8.4% to 4.82 ± 1.38% (judged morphologically, *n* = 4), implicating the activation of caspases in antagonist G-induced apoptosis.

Antagonist G-induced apoptosis was blocked by co-incubation with the ROS scavenger N-acetyl cysteine (10 mM n-AC) and by incubation under anoxic conditions (Figure 1B). This extends our previous work and suggests that similar to JNK activation (MacKinnon et al, 1999), antagonist G-induced apoptosis occurs via an oxidant-dependent mechanism in SCLC cells.

### Antagonist G-induced generation of intracellular ROS in SCLC cells is cell type-specific

The finding that the apoptotic effect of antagonist G was abrogated by n-AC and anoxia suggests that antagonist G requires the

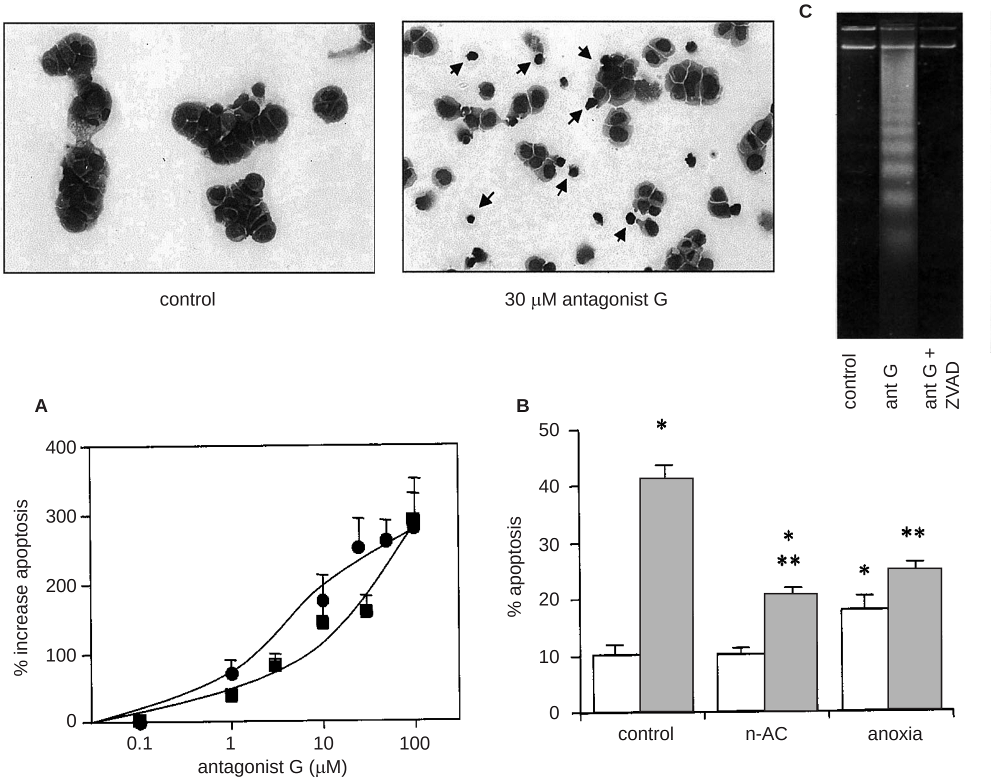

**Figure 1** Effect of antagonist G on SCLC cell apoptosis. (**A**) SCLC cells H69 (●) and H510 (■) were washed and incubated in serum-free quiescent medium with antagonist G or diluent ($H_2O$) for 24 h at 37°C. Apoptosis was assessed morphologically using May–Grünwald–Giemsa stain as described in Methods. The data is expressed as % increase in apoptosis and represents the mean ± SEM of four independent experiments performed in triplicate. Representative photomicrographs (upper panel) of histologically stained H69 cells treated with diluent (left) or 30 μM antagonist G (right) for 24 h, showing the presence of apoptotic bodies. (**B**) Antagonist G-induced apoptosis is inhibited by anoxia and n-acetyl cysteine. H69 cells cultured in SITA medium were quiesced overnight in serum-free medium, washed and incubated ($1 \times 10^6$ cells $ml^{-1}$) in the absence (open bars) or presence (closed bars) of 25 μM antagonist G with or without 10 mM n-acetyl cysteine (n-AC) for 24 h at 37°C. H69 cells were made anoxic by incubation in a MK3 anaerobic incubator (0% $O_2$) at 37°C for 24 h. Apoptosis was assessed morphologically using May–Grünwald–Giemsa stain as described in Methods. The results are expressed as % apoptotic cells and represent the mean ± SEM of three–four separate experiments performed in duplicate. (**C**) Representative DNA ladder showing the effects of 25 μM antagonist G with or without 100 μM ZVAD-fmk. *Statistically different from control; **statistically different from corresponding antagonist G control (*P* < 0.05 ANOVA)

*© 2000 Cancer Research Campaign* 

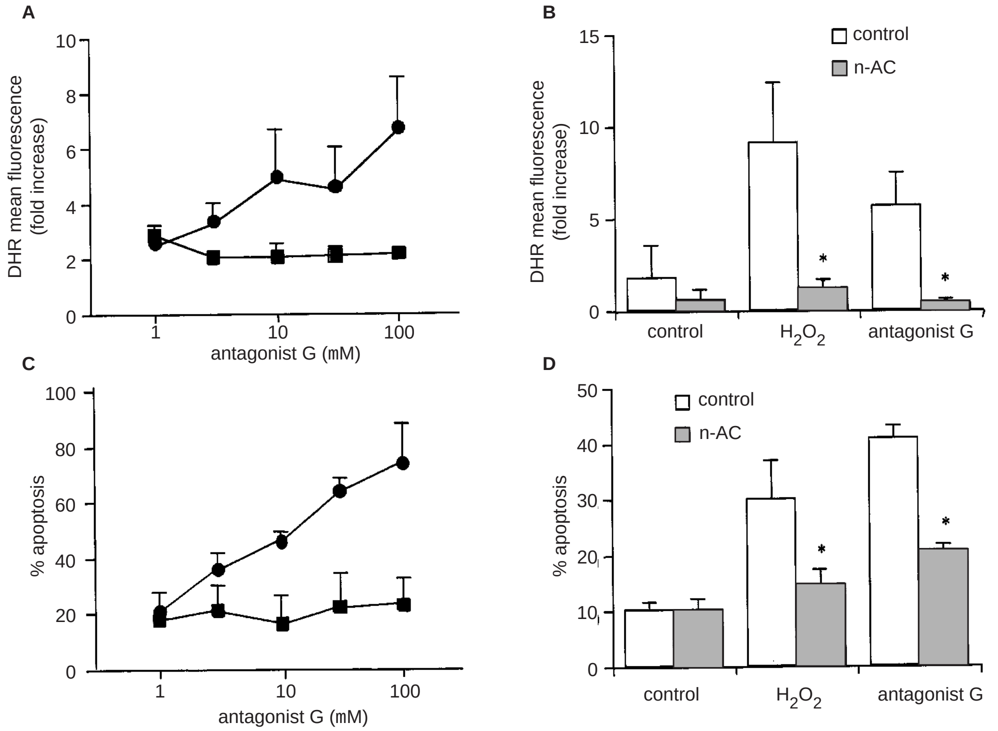

**Figure 2** (**A**) Antagonist G generates intracellular ROS in SCLC but not CHO-K1 cells. SCLC H69 (●) and CHO-K1 (■) cells were washed and incubated ($10^6$ cells $ml^{-1}$) with 1 μM dihydrorhodamine (DHR) for 5 min at 37°C prior to the addition of antagonist G for a further 10 min at 37°C. Fluorescence was measured by flow cytometry using an EPICS Profile II flow cytometer (Coulter Electronics, Luton, UK.). The data represent the mean ± SEM of four experiments carried out in duplicate. (**B**) Effect of NAC on ROS generation. DHR-loaded H69 cells were preincubated with 5 mM n-AC for 20 min at 37°C. Cells were then incubated in the absence or presence of 200 μM $H_2O_2$ or 50 μM antagonist G. Fluorescence was measured as in (**C**). The data are expressed as % increase in DHR fluorescence and represent the mean ± SEM of at least three independent experiments carried out in duplicate. (**C**) Effect of antagonist G on H69 and CHO-K1 cell apoptosis. H69 SCLC cells were treated as in Figure 1 and incubated with annexin V as described in Methods. CHO-K1 cells were treated 50 μM antagonist G for 24 h in DMEM containing 0.1% fetal bovine serum. The percentage of annexin V-positive (apoptotic cells) was assessed by flow cytometry. The data represent the mean ± SEM of at least three independent experiments carried out in duplicate. (**D**) Effect of n-AC on antagonist G-induced apoptosis. H69 cells were treated as in (**B**) in the presence or absence of 5 mM n-AC and/or 50 μM antagonist G. Apoptosis was assessed by annexin-V positivity and is expressed as % apoptosis. The data represent the mean ± SEM of at least three independent experiments carried out in duplicate. *Statistically different from control ($P < 0.05$ ANOVA)

generation of ROS to induce apoptosis in SCLC cells. This was formally examined by measuring intracellular ROS generation. Cells were preloaded with the cell-permeable oxidant-sensitive fluorescent dye dihydrorhodamine (DHR) and ROS generation followed as an increase in fluorescence measured by flow cytometry. $H_2O_2$ (200 μM) increased DHR fluorescence, causing a 5.1-fold increase in H69 cells (from 1.8 ± 0.09 to 9.2 ± 3.2 mean fluorescence units; $n = 4$, Figure 2B). Similar results were shown in the H510 cell line (data not shown). At concentrations which induce apoptosis, antagonist G produced a dose-dependent increase in DHR fluorescence (Figure 2A) causing a 6.2-fold increase with 100 μM antagonist G in SCLC cells. The finding that this effect was evident within 15 min exposure to antagonist G and well before morphological changes were apparent suggests that this is a primary event involved in a pro-apoptotic pathway in SCLC cells. This was emphasized by the finding that although the antagonist G-induced increase in DHR fluorescence and apoptosis was blocked by n-acetyl cysteine (Figure 2B and 2D), only the apoptotic effect was inhibited by the caspase inhibitor ZVAD-fmk (Figure 1C). ZVAD-fmk had no effect on antagonist-G induced DHR fluorescence (data not shown). Antagonist G was not acting as an oxygen donor itself as it had no effect on cytochrome c oxidation in vitro (data not shown). However, antagonist G at concentrations which increased ROS generation in the SCLC cell lines had no effect on DHR fluorescence in CHO-K1 cells (Figure 2A). In addition, antagonist G did not induce apoptosis in CHO-K1 cells (Figure 2C). These experiments indicate that the oxidant-dependent apoptotic effect of antagonist G is not non-specific and generally cytotoxic, but is cell type-specific.

### Antagonist G depletes cellular glutathione

In keeping with a ROS-dependent mechanism, antagonist G (30 μM) increased peroxyl radical generation by 112% in H510 cells (Figure 3C) and depleted the levels of GSH by 38% in H510 cells. This reduction in cellular GSH levels was similar to that observed with a maximal concentration of L-buthionine-(*SR*)-sulfoximine (BSO, 0.1 mM), an inhibitor of the GSH synthesizing enzyme glutamylcysteine synthetase (Figure 3A).

Ceramide is an important lipid second messenger which has been implicated in the apoptotic response to a wide variety of cellular stresses including oxidant stress (Haimovitz-Friedman et

 *© 2000 Cancer Research Campaign*

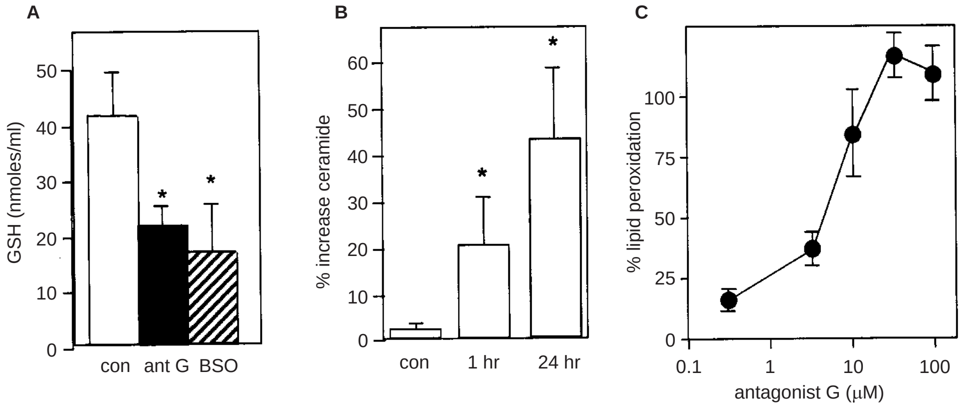

**Figure 3** (**A**) Effect of antagonist G on GSH levels in SCLC cells. Quiescent cultures of H510 cells ($5 \times 10^6$ cells $ml^{-1}$) were incubated in serum-free medium in the presence of 50 μM antagonist G (ant G) or 0.1 mM L-buthionine-[*SR*]-sulphoximine (BSO) for 24 h at 37°C. Total and oxidized GSH was measured from cellular extracts as described in Methods. The data are expressed as nmole GSH $ml^{-1}$ extract and represent the mean ± SEM of three–four independent experiments performed in triplicate. (**B**) Ceramide production. SCLC H510 cells in SITA medium were incubated with 50 μM antagonist G for 1 h or 24 h at 37°C. Ceramide from extracted lipids was measured by the DAG kinase assay as described in Methods. The results are expressed as % increase above control and represent the mean ± SEM of three experiments performed in triplicate. (**C**) Lipid peroxidation. SCLC H510 cells in serum-free media were incubated with increasing concentrations of antagonist G for 24 h. Lipid peroxides were assayed as thiobarbituric acid reactive substances (TBARS) as described in Methods. The data are expressed as % increase above control and represent mean ± SD of two independent experiments performed in triplicate. *Statistically different from control ($P < 0.05$ ANOVA)

al, 1994; Verheij et al, 1996). The JNK cascade is one of the downstream signalling pathways activated by ceramide. We show that antagonist G (50 μM) increases ceramide levels in SCLC cells after 1 h and 24 h treatment (Figure 3B).

### Antagonist G stimulates JNK and AP-1 activity

Figure 4A shows that antagonist G (25 μM) and $H_2O_2$ (200 μM) stimulate JNK activity in H69 cells. The transcription factor AP-1 has been associated with apoptosis (Staal et al, 1990; Devary et al, 1991; Jarpe et al, 1998*b*) and its transcriptional activity is increased by JNK (Staal et al, 1990; Devary et al, 1991). Figure 4B shows that antagonist G (10 μM) increased AP-1 activation by 61 ± 14% ($n = 4$) compared to an 84 ± 18% increase induced by $H_2O_2$ (200 μM, $n = 4$, Figure 4B). The specificity was confirmed by the inhibition of binding by unlabelled AP-1 but not NFκβ (Figure 4B). Thus, antagonist G stimulates JNK and AP-1 activation via a redox-sensitive mechanism in SCLC cells.

### Antagonist G sensitizes SCLC cells to chemotherapy

Previous work has shown that modulation of the redox balance by altering the levels of GSH changes the response of cells to cytotoxic drugs (Russo et al, 1984). Moreover, the levels of GSH have been shown to increase in SCLC and NSCLC cells as they become resistant to chemotherapy (Ikegaki et al, 1994). The ability of antagonist G to increase ROS and deplete GSH suggests that antagonist G may sensitize SCLC cells to chemotherapeutic agents. We therefore examined whether antagonist G could potentiate growth inhibition induced by the chemotherapeutic agent etoposide.

Etoposide (0.3 μg $ml^{-1}$), or antagonist G (5 μM) had no effect on H69 SCLC cell growth individually, however, when added in combination, H69 SCLC cell proliferation was inhibited by 24% (Figure 5A). 50 μM antagonist G did inhibit growth by 59% and this was potentiated to 79% inhibition in the presence of 0.3 μg $ml^{-1}$ etoposide. The effect of antagonist G on etoposide-induced growth inhibition was more than additive and was synergistic showing an augmentation of the maximum response to etoposide rather than a change in affinity (Figure 2B). These data show that antagonist G can sensitize SCLC cells to etoposide-induced growth inhibition, and suggest an additional role for antagonist G to be co-administered with standard chemotherapy in the treatment of SCLC.

### DISCUSSION

We demonstrate that altering the redox status of the cell toward a more reduced environment modulates the pro-apoptotic effect of antagonist G. This was demonstrated from observations that the induction of apoptosis by antagonist G is inhibited under anoxic conditions and that antagonist G directly increases the generation of ROS within the cell. Antagonist G is not an oxygen donor itself as it has no effect on isolated cytochrome c activity in vitro. We suggest that the ability of antagonist G to stimulate intracellular ROS is not a general cytotoxic effect as it is unable to increase ROS generation and induce apoptosis in CHO-K1 cells and in isolated human neutrophils (unpublished observations). Therefore, it appears that antagonist G increases cellular ROS specifically in SCLC cells. This, coupled with the finding that anoxia and N-acetyl cysteine block the ability of antagonist G to induce apoptosis, suggests that this is an important part of its pro-apoptotic mechanism of action, which occurs upstream of caspase activation.

The transcription factor AP-1 is regulated by JNK and its transcriptional activity is increased by ROS in many cell types (Staal et al, 1990; Devary, 1991; Buttke and Sandstrom, 1994; Cossarizza et al, 1995; Lo and Cruz, 1995). Although increased JNK and AP-1 activity is associated with cellular proliferation in many cell types, in neuronal cells such as PC12 and in certain models of stress-induced apoptosis, increased activity of JNK and AP-1 are associated with apoptosis (Verheij et al, 1996; Jarpe

© 2000 Cancer Research Campaign

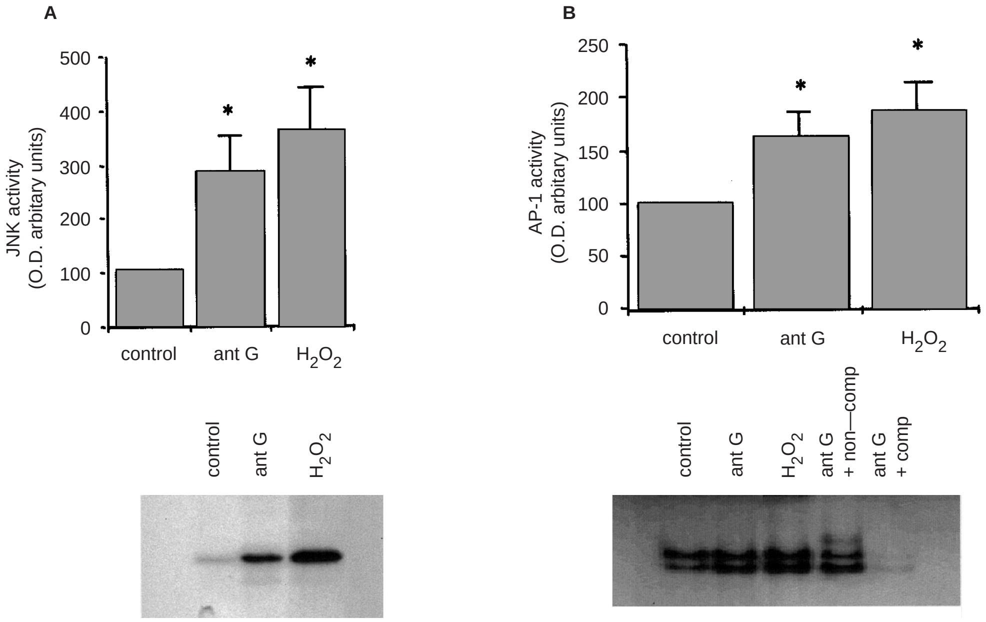

**Figure 4** Regulation of JNK and AP-1 by antagonist G. (**A**) JNK activity. Quiescent H69 cells were incubated ($1 \times 10^7$ cells $ml^{-1}$) in PBS with 25 μM antagonist G (ant G) or 200 μM $H_2O_2$. JNK activity was assessed from JNK1 immunoprecipitates as described in Methods. Activity was compared with the control value set at 100%. The data represent the mean and SD of two determinations. A representative autoradiogram is shown. (**B**) AP-1 activity. SCLC H69 cells were treated with 10 μM antagonist G (ant G) or 200 μM $H_2O_2$ for 30 min at 37°C. Nuclear extracts were isolated and incubated in the presence of labelled AP-1 oligonucleotide. DNA binding to AP-1 was analysed by EMSA. The specificity of binding is shown by incubation with 100-fold excess of either unlabelled AP-1 oligonucleotide (comp) or unlabelled NFκβ oligonucleotide (non-comp). Densitometric quantitation of AP-1 binding is shown in the upper panel. Activation was compared with the control value set at 100%. Data are expressed as mean ± SEM of relative intensity of bands from three separate experiments

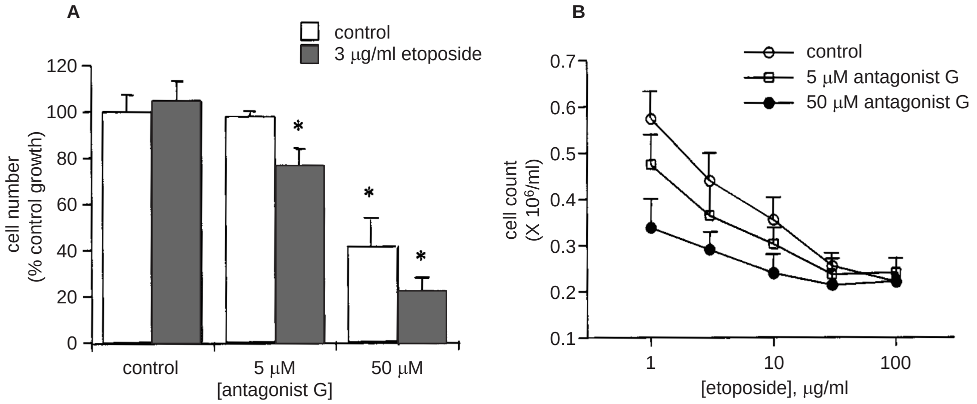

**Figure 5** Effect of antagonist G on etoposide-induced growth inhibition. (**A**) H69 cells were incubated with etoposide (0.3 μg $ml^{-1}$) in the absence or presence of 5 μM or 50 μM antagonist G. Total cell number was determined at day 9. The data are expressed as % control cell number and represent the mean ± SEM of three independent experiments. (**B**) H69 cells were incubated with etoposide (1–100 μg $ml^{-1}$) in the absence (□) or presence of 5 μM (●) or 50 μM (■) antagonist G. Total cell number was determined at day 9. The data are expressed as cell number $ml^{-1}$ and represent the mean ± SEM of three independent experiments et al, 1998*b*; Le-Niculescu et al, 1999). This study shows that antagonist G increases AP-1 activity at concentrations which induce apoptosis and activate JNK in SCLC cells, and gives further evidence for the importance of ROS in the mediation of antagonist G's effects.

We show that antagonist G depletes the levels of the major cellular antioxidant glutathione. There is evidence from other systems that such a response can lead to a stimulation of the stress pathway and programmed cell death. For example it has been shown that glutathione directly inhibits sphingomyelinase (Liu and Hannun, 1997), thereby reducing the levels of ceramide, an important inducer of apoptosis in many cells (Haimowitz-Friedman et al, 1994; Pena et al, 1997). In turn, ceramide stimulates the electron transport chain of mitochondria, leaking free

 *© 2000 Cancer Research Campaign*

electrons to generate superoxide anion (Zamzami et al, 1995) which leads to a depletion of GSH and an increase in the generation of ROS (Quillet-May et al, 1997). In Jurkat T cells, the cellular GSH level was suggested to be critical for ROS activation of JNK in a pathway involving ras or other GTPases such as rac1 or cdc 42 as redox sensors (Lander et al, 1996). Therefore, it appears that the level of GSH within the cell plays an important role in modulating stress responses leading to apoptosis and cell death.

Small cell lung cancer is characteristically resistant to oxidative damage and lipid peroxidation (Galeotti et al, 1991). Patients with lung cancer have increased levels of antioxidant GSH in the lungs, which renders them more resistant to oxidant-or chemotherapy-induced cell death (Russo et al, 1984; Melloni et al, 1996). Moreover, modulation of GSH levels has been shown to change the response of cells to cytotoxic drugs and ionizing radiation (Carmichael et al, 1988). L-buthionine-*SR*-sulfoximine (BSO), an inhibitor of γ-glutamylcysteine synthetase, has undergone Phase I clinical development to lower elevated GSH in patients with lung cancer (Gallo et al, 1995), however, there is no compound currently available which employs the dual function of increasing ROS and depleting GSH with the potential to cause persistent activation of JNK and apoptosis. Here we show for the first time an explicitly novel mechanism of action of antagonist G to induce apoptosis through a redox-sensitive pathway. We also show that antagonist G (at concentrations achieved clinically in the Phase I studies (Clive et al, 1999) is able to synergistically sensitize SCLC cells to etoposide at concentrations which were otherwise ineffective. This will have important implications for the future clinical use of antagonist G as tumours progress from chemosensitive to chemoresistant phenotypes. This suggests a role for antagonist G not only as a sole treatment for SCLC but as an additional treatment with chemotherapy. Antagonist G may therefore have a dual beneficial role in SCLC and may also be of benefit in a variety of other tumours by sensitizing them to subsequent chemotherapy.

### ACKNOWLEDGEMENTS

This work was supported in part by the Scottish Hospitals Endowment Research Trust (Grant No. SHERT 1441) and the Melville Trust for the Care and Cure of Cancer.

### REFERENCES

Bligh EG and Dyer WJ (1959) A rapid method of total lipid extraction and purification. *Can J Biochem Physiol* **37**: 911–917

Bush RS, Jenkin RDT, Allt WEC, Beale FA, Bean H, Dembo AJ and Pringle JF (1978) Definitive evidence for hypoxic cells influencing cure in cancer therapy. *Br J Cancer* **37**: 302–306

Butterfield L, Storey B, Maas L and Heasley LE (1997) c-jun $NH_2$-terminal kinase regulation of the apoptotic response of small cell lung cancer cells to ultraviolet radiation. *J Biol Chem* **272**: 10110–10116

Buttke TM and Sandstrom PA (1994) Oxidative stress as a mediator of apoptosis. *Immunol Today* **15**: 7–11

Carmichael J, Mitchell JB, Friedman N, Gazdar AF and Russo A (1988) Glutathione and related enzyme activity in human cancer cell lines. *Br J Cancer* **58**: 437–440

Clive S, Jodrell DI, MacLellan A, Webb DJ, Young A, Miller M, Cummings J and Smyth J (1999) Phase I, pharmacokinetic (PK) and pharmacodynamic (PD) study of antagonist G, a broad spectrum neuropeptide growth factor antagonist. *Proc Am Assol Cancer Res* **40**: P156

Cossarizza A, Franceschi C, Monti D, Salvioli S, Bellesia E, Rivabene R, Biondo L, Rainaldi G, Tinari A and Malorni W (1995) Protective effect of N-acetylcysteine in tumor necrosis factor-alpha-induced apoptosis in U937 cells: the role of mitochondria. *Exp Cell Res* **220**: 232–240

Coso OA, Chiariello M, Kalinec G, Kyriakis JM, Woodgett J and Gutkind JS (1995) Transforming G protein coupled receptors potently activate JNK (SAPK). *J Biol Chem* **270**: 5620–5624

Cuttitta F, Carney J, Mulshine J, Moody TW, Fedorko J, Fischler A and Minna JD (1985) Bombesin-like peptides can function as autocrine growth factors in human small-cell lung cancer. *Nature* **316**: 823–826

Devary Y, Gottlieb RA, Lau LF and Karin M (1991) Rapid and preferential activation of the c-jun gene during the mammalian UV response. *Mol Cell Biol* **11**: 2804–2811

Friesen C, Fulda S and Debatin KM (1999) Cytotoxic drugs and the CD95 pathway. *Leukemia* **11**: 1854–1858

Galeotti T, Masotti L, Borello S and Casali E (1991) Oxy-radical metabolism and control of tumour growth. *Xenobiotica* **21**: 1041–1051

Gallo JM, Brennan J, Hamilton TC, Halbherr T, Laub PB, Ozols RF and O'Dwyer PJ (1995) Time-dependent pharmacodynamic models in cancer chemotherapy: Population pharmacodynamic model for glutathione depletion following modulation by buthionine sulfoximine (BSO) in a Phase I trial of melphalan and BSO. *Cancer Res* **55**: 4507–4511

Haimovitz-Friedman A, Kan C, Ehleiter D, Persaud RS, McLoughlin M, Fuks Z and Kolesnick RN (1994) Ionising radiation acts on cellular membranes to generate ceramide and initiate apoptosis. *J Exp Med* **180**: 525–535

Ihde DC (1992) Chemotherapy of lung cancer. *N Engl J Med* **327**: 1434–1441

Ikegaki N, Katsumata M, Minna J and Tsujimoto Y (1994) Expression of bcl-2 in small cell lung carcinoma cells. *Cancer Res* **54**: 6–8

Jarpe MB, Knall C, Mitchell FM, Buhl A, Duzic E and Johnson GL (1998*a*) [D-$Arg^1$, D-$Phe^5$, D-$Trp^{7,9}$, $Leu^{11}$} substance P acts as a biased agonist towards neuropeptide and chemokine receptors. *J Biol Chem* **273**: 3097–3104

Jarpe MB, Widmann C, Knall C, Schlesinger TK, Gibson S, Yujiri T, Fanger GR, Gelfand EW and Johnson GL (1998*b*) Anti-apoptotic versus pro-apoptotic signal transduction: Checkpoints and stop signs along the road to death. *Oncogene* **17**: 1475–1482

Jayadev S, Linardic CM and Hannun YA (1994) Identification of arachidonic acid as a mediator of sphingomyelin hydrolysis in response to tumor necrosis factor α. *J Biol Chem* **269**: 5757–5763

Jonkman-de Vries JD, Rosing H, Talsma H, Henrar REC Kettenes-van den Bosch IJ, Bult A and Beijnen JH (1998) Pharmaceutical development of a parenteral lyophilised formulation of the investigational antitumor neuropeptide antagonist [Arg6, D-Trp7,9, MePhe8]-substance P (6–11). *Invest New Drugs* **16**: 99–111

Kerr JFK, Wyllie AH and Currie AH (1972) Apoptosis, a basic biological phenomenon with wider implications in tissue kinetics. *Br J Cancer* **26**: 239–245

Kyprianou N, English HF, Davidson NE and Isaacs JT (1992) Programmed cell death during regression of the MCF-7 human breast cancer following estrogen ablation. *Cancer Res* **51**: 162–166

Lander HM, Jacovina AT, Davis RJ and Taurus JM (1996) Differential activation of mitogen-activated protein kinases by nitric oxide related species. *J Biol Chem* **271**: 19705–19709

Langdon SP, Cummings J and Smyth JF (1994) Antagonists of mitogenic peptides. *Cancer Topics* **9**: 10–13

Le-Niculescu H, Bonfoco E, Kasuya Y, Claret FX, Green DR and Karin M (1999) Withdrawal of survival factors results in activation of the JNK pathway in neuronal cells leading to Fas ligand induction and cell death. *Mol Cell Biol* **1**: 751–763

Liu B and Hannun YA (1997) Inhibition of the neutral magnesium-dependent sphingomyelinase by glutathione. *J Biol Chem* **272**: 16281–16287

Lo YYC and Cruz TF (1995) Involvement of reactive oxygen species in cytokine and growth factor induction of c-fos expression in chondrocytes. *J Biol Chem* **270**: 11727–11730

MacKinnon AC, Armstrong RA, Waters C, Cummings J, Smyth JF, Haslett C and Sethi T (1999) [$Arg^6$, D-$Trp^{7,9}$, $N^{me}Phe^8$]-substance P (6–11) activates JNK and induces apoptosis in small cell lung cancer cells via an oxidant dependent mechanism. *Br J Cancer* **80**: 1026–1034

Melloni B, Lefebvre M, Bonnaud F, Vergnenegre A, Grossin L, Rigaud M and Cantin A (1996) Antioxidant activity in bronchoalveolar lavage fluid in patients with lung cancer. *Am J Respir Crit Care Med* **154**: 1706–1711

Mitchell FM, Heasley LE, Qian NX, Zamarripa J and Johnson GL (1995) Differential modulation of bombesin-stimulated phospholipase Cb and mitogen-activated protein kinase activity by [D-$Arg^1$, D-$Phe^5$, D-$Trp^{7,9}$, $Leu^{11}$]substance P. *J Biol Chem* **270**: 8623–8628

Moody TW, Pert CB, Gazdar AF, Carney DN and Minna JD (1981) High levels of intracellular bombesin characterize human small-cell lung carcinoma. *Science* **214**: 1246–1248

© 2000 Cancer Research Campaign

Ohkawa H, Ohishi N and Yagi K (1979) Assay of lipid peroxides in animal tissues by thiobarbituric acid reaction. *Anal Biochem* **95**: 351–358

Pena LA, Fuks Z and Kolesnick R (1997) Stress-induced apoptosis and the sphingomyelin pathway. *Biochem Pharmacol* **53**: 615–621

Quillet-May A, Jaffrezou JP, Mansat V, Bordier C, Naval J and Laurent G (1997) Implication of mitochondrial hydrogen peroxide generation in ceramide-induced apoptosis. *J Biol Chem* **272**: 21388–21395

Russo A, Mitchell JB, McPherson S and Friedman N (1984) Alteration of bleomycin cytotoxicity by glutathione depletion or elevation. *Int J Radiat Oncol Biol Phys* **10**: 1675–1679

Seckl MJ, Newman RH, Freemont PS and Rozengurt E (1995) Substance P-related antagonists inhibit vasopressin and bombesin but not 5′-3-O-(thio)triphosphate-stimulated inositol phosphate production in Swiss 3T3 cells. *J Cell Physiol* **163**: 87–95

Seckl MJ, Higgins T and Rozengurt E (1996) [D-Arg$^{1}$, D-Trp$^{5,7,9}$, Leu$^{11}$]-substance P coordinately and reversibly inhibits bombesin and vasopressin-induced signal transduction pathways in Swiss 3T3 cells. *J Biol Chem* **271**: 29453–29460

Seckl MJ, Higgins T, Widmer F and Rozengurt E (1997) [D-Arg$^{1}$, D-Trp$^{5,7,9}$, Leu$^{11}$ ]-substance P: a novel potent inhibitor of signal transduction and growth in vitro and in vivo in small cell lung cancer cells. *Cancer Res* **57**: 51–54

Sethi T and Rozengurt E (1991) Multiple neuropeptides stimulate clonal growth of small cell lung cancer: effects of bradykinin, vasopressin, cholecystokinin, galanin, and neurotensin. *Cancer Res* **51**: 3621–3623

Sethi T, Langdon SP, Smyth JS and Rozengurt E (1992) Growth of small cell lung cancer cells, stimulation by multiple neuropeptides and inhibition by broad spectrum antagonists in vitro and in vivo. *Cancer Res* **52**: 2737s–2742s

Smyth JF, Fowlie SM, Gregor A, Crompton GK, Busutill A, Leonard RCF and Grant IWB (1986) The impact of chemotherapy on small cell carcinoma of the bronchus. *Q J Med* **61**: 969–976

Staal FJ, Roederer M, Herzenberg LA and Herzenberg LA (1990) Intracellular thiols regulate activation of nuclear factor kappa B and transcription of human immunodeficiency virus. *Proc Natl Acad Sci USA* **87**: 9943–9947

Tallet A, Chilvers ER, Hannah S, Dransfield I, Lawson MF, Haslett C and Sethi T (1996) Inhibition of neuropeptide-stimulated tyrosine phosphorylation and tyrosine kinase activity stimulates apoptosis in small cell lung cancer cells. *Cancer Res* **56**: 4255–4263

Tietze F (1969) Enzymic method for quantitative determination of nanogram amounts of total and oxidised glutathione: Applications to mammalian blood and other tissues. *An Biochem* **27**: 502–522

Verheij M, Bose R, Lin XH, Yao B, Jarvis WD, Grant S, Birrer MJ, Szabo E, Zon LI, Kyriakis JM, Haimovitz-Friedman A, Fuks Z and Kolesnick RN (1996) Requirement for ceramide-initiated SAPK/JNK signalling in stress-induced apoptosis. *Nature* **380**: 75–79

Woll PJ and Rozengurt E (1988) [D-Arg$^{1}$, D-Phe$^{5}$, D-Trp$^{7,9}$, Leu$^{11}$]-substance P a potent bombesin antagonist in murine Swiss 3T3 cells, inhibits the growth of small cell lung cancer in vitro. *Proc Natl Acad Sci USA* **85**: 1859–1863

Woll PJ and Rozengurt E (1990) A neuropeptide antagonist that inhibits the growth of small cell lung cancer in vitro. *Cancer Res* **50**: 3868–3873

Zamzami N, Marchetti P, Castedo M, Decaudin D, Macho A, Hirch T, Susin SA, Petit PX, Mignotte B and Kroemer G (1995) Sequential reduction of mitochondrial transmembrane potential and generation of reactive oxygen species in early programmed cell death. *J Exp Med* **182**: 367–377

© 2000 Cancer Research Campaign
